# The Network of Interactions Among Porcine Reproductive and Respiratory Syndrome Virus Non-structural Proteins

**DOI:** 10.3389/fmicb.2018.00970

**Published:** 2018-05-14

**Authors:** Hao Nan, Jixun Lan, Mengmeng Tian, Shan Dong, Jiao Tian, Long Liu, Xiaodong Xu, Hongying Chen

**Affiliations:** ^1^College of Life Sciences, Northwest A&F University, Yangling, China; ^2^School of Basic Medical Sciences, Hubei University of Medicine, Shiyan, China; ^3^Shaanxi Key Laboratory of Agricultural and Environmental Microbiology, College of Life Sciences, Northwest A&F University, Yangling, China

**Keywords:** porcine reproductive and respiratory syndrome virus, non-structural proteins, protein–protein interaction, yeast two-hybrid, bimolecular fluorescence complementation assay, pull-down assay

## Abstract

The RNA synthesis of porcine reproductive and respiratory syndrome virus (PRRSV), a positive-strand RNA virus, is compartmentalized in virus-induced double-membrane vesicles where viral proteins and some cellular proteins assemble into replication and transcription complexes (RTCs). The viral replicase proteins are the major components of the RTCs but the physical associations among these non-structural proteins (nsps) remain elusive. In this study, we investigated the potential interactions between PRRSV nsps by yeast two-hybrid (Y2H), bimolecular fluorescence complementation (BiFC) and pull-down assays. Our analyses revealed a complex network of interactions involving most of PRRSV nsps. Among them, nsp9 and nsp12 were identified as the hubs of the nsp interactome; transmembrane proteins nsp2 and nsp5 both interacted with nsp3, indicating that the three membrane-bound proteins might bind together to form the scaffold to support the association of RTCs with the intracellular membrane. The PRRSV nsp interactions identified in this study may provide valuable clues for future researches on the RTC formation and function.

## Introduction

Porcine reproductive and respiratory syndrome virus (PRRSV), the causative agent of porcine reproductive and respiratory syndrome (PRRS) which has caused great economic losses to the pig industry, belongs to the family Arteriviridae in the order Nidovirales. It is an enveloped virus encapsulating a positive-stranded RNA genome of about 15 kb. The genome contains at least 11 open reading frames (Fang et al., 2012; Kappes and Faaberg, 2015; Li et al., 2015). ORF1a and ORF1b at the 5′-proximal three quarters of the genome encode long polyproteins pp1a and pp1ab which are subsequently cleaved by viral proteases to generate functional non-structural proteins (nsps), and the rest 3′- proximal part of the genome consists of ORFs encoding viral structural proteins. A short alternative ORF that overlaps ORF1a in the +1/-2 frame was recently identified, and the ORF could be expressed to yield a transframe protein nsp2TF and a truncated nsp2 variant nsp2N by programmed ribosomal frameshifting (PRF) mechanism (Fang et al., 2012).

Among the nsps encoded by ORF1a, nsp1α/β and nsp2 contain papain-like proteinase (PLP) domains and are rapidly released by autoproteolytic cleavage of the polyproteins after their synthesis. nsp4 is the main proteinase that is responsible for the subsequent processing of the remaining polyproteins via a major processing pathway with nsp2 acting as a cofactor and a minor processing pathway in the absence of nsp2 (Li et al., 2015). Hydrophobic nsp2, nsp3, and nsp5 containing predicted membrane-spanning domains are thought to be involved in membrane modification and formation of double membrane vesicles (DMVs), a typical structure induced by arterivirus infection. Nsp6 and nsp8 are short peptides containing only 16 and 45 amino acid residues, respectively, and their functions in virus infection remain unknown. With an internal cleavage site, nsp7 can be further cleaved into nsp7α and nsp7β, however, only cleaved nsp7α was detectable in PRRSV infected cells by western blot and radioimmunoprecipitation (Li et al., 2012; Chen et al., 2017). The specific function of nsp7 is also unclear although structure-based reverse genetics studies have demonstrated that it plays an important role in arterivirus RNA synthesis (Manolaridis et al., 2011; Zhang et al., 2013).

ORF1b is the most conserved region of arterivirus genomes. It encodes four more nsps including the viral RNA dependent RNA-polymerase (RdRp) nsp9, RNA helicase nsp10, endoribonuclease (NendoU) nsp11 and nsp12 with unknown function (Fang and Snijder, 2010). As the key enzyme for viral RNA synthesis, nsp9 is considered to be a core component of the viral replication and transcription complex (RTC) (Knoops et al., 2012). The RdRp domain locates in the C-terminal part of nsp9, and the N-terminal part of the protein is identified as a nidovirus RdRp-associated nucleotidyltransferase domain (Lehmann et al., 2015).

During arterivirus infection, most nsps have been found to localize to membranes in the perinuclear region of the infected cell and assemble into a membrane-associated viral RTC (Pedersen et al., 1999; Knoops et al., 2012). The transmembrane proteins nsp2, nsp3, and nsp5 are believed to anchor the RTC to intracellular membranes and transform them into DMVs. Studies have found that arterivirus nsp2 interacts with nsp3 and the expression of the two nsps induces the formation of structures similar to the infection-induced DMVs (Snijder et al., 2001). PRRSV nsp1α, nsp1β have also been reported to directly interact to nsp2 (Song et al., 2016). Our previous study have identified the binding of nsp7α to the viral RdRp (Chen et al., 2017). By immunofluorescence analysis, nsp1β, nsp2, nsp4, nsp7α, nsp7β, and nsp8 have been observed to co-localize in distinct punctate foci in the perinuclear region of the cell, which is the site of viral RNA synthesis during the early stages of infection (Li et al., 2012). However, how these nsps are associated and involved in the formation of RTC in DMVs remains to be elucidated.

In this study, we investigated the interactions among the PRRSV non-structural proteins by yeast two-hybrid (Y2H) and bimolecular fluorescence complementation (BiFC) assays, and verified some of the identified interactions by pull-down assays. We observed a complex network of interactions involving most of PRRSV nsps, which may provide a framework for future research on the RTC formation and function.

## Materials and Methods

### Virus Genes and Sequences

The replicon FL-12 (Truong et al., 2004) of PRRSV North American strain NSVL 97-7895 (GenBank accessionno.AY545985.1) was used as the template for the amplification of nsp genes by PCR. The nsps used in this study and their known or predicted functions are listed in **Table 1**.

**Table 1:** porcine reproductive and respiratory syndrome virus (PRRSV) non-structural proteins (NSPs) used in this study and their known or predicted functions.

Name	N- and C-terminal residues of each polypeptide used in this study	Known or predicted functions
NSP1α	Met1–Met180 (180 aa)	Accessory protease Papain-like cysteine protein protease-α; viral transcription factor; potential IFN antagonist
NSP1β	Ala181–Gly383 (203 aa)	Accessory protease Papain-like cysteine protein protease-β; potential IFN antagonist
NSP2	Ala384–Gly1579 (1196 aa) (for BiFC) Ala384–Pro1197(814aa) (for Y2H)	Accessory cysteine protease; transmembrane protein involved in membrane modification; a member of the ovarian tumor domain (OTU) family of deubiquitinating enzymes; potential IFN antagonist
NSP3	Gly1580–Glu1809 (230 aa) (for BiFC) Try1602–Val1652 (51aa) + Arg1749–Glu1809 (61aa) (for Y2H and pull-down)	Transmembrane protein involved in membrane modification
NSP4	Gly1810–Glu2013 (204 aa)	A 3C-like serine proteases; potential IFN antagonist; apoptosis inducer
NSP5	Gly2014–Glu2183 (170 aa) (for BiFC) Gly2033–Arg2091(59aa) + Ala2111–Arg2131(21aa) (for Y2H and pull-down)	Transmembrane protein involved in membrane modification; suppressor of JAK/STAT3 pathway
NSP7α	Ser2200–Glu2348 (149 aa)	Unknown
NSP7β	Asn2349–Glu2458 (110 aa)	Unknown
NSP8	Ala2459–Cys2503 (45 aa)	Unknown
NSP9	Ala2459–Glu3143 (685 aa)	RNA-dependent RNA polymerase
NSP10	Gly3144–Glu3584 (441 aa)	NTPase; RNA helicase
NSP11	Gly3585–Glu3807 (223 aa)	Endoribonuclease (NendoU); potential IFN antagonist
NSP12	Gly3808–Asn3960 (153 aa)	Potential IFN inhibitor; inducer of proinflammatory cytokines

### Yeast Two-Hybrid (Y2H) Screening of Nsp Interactions

Each of the NSP gene segments was produced by polymerase chain reaction (PCR) using the corresponding primers listed in Supplementary Table S1, and then inserted into pGBKT7 and pGADT7, respectively. For transmembrane proteins nsp2, nsp3 and nsp5, the transmembrane domains identified by TMHMM Server v. 2.0 were deleted from the genes by PCR. The inserts in the constructs were verified by DNA sequencing.

The pGADT7-nsp and pGBKT7-nsp constructs were co-transformed in pairs into *Saccharomyces cerevisiae* (yeast) strain Y2HGold using the Yeastmaker Yeast Transformation System kit (Clontech, United States) according to the manufacturer’s instruction. Selection of co-transformed colonies was performed on selective medium lacking leucine and tryptophan. Protein interactions were examined by growing on minimal synthetic medium lacking adenine, histidine, leucine, and tryptophan (SD/-Ade/-His/-Leu/-Trp).

### Bimolecular Fluorescence Complementation (BiFC) Assay

The full-length nsp gene fragments used in BiFC assays (listed in **Table 1**) were amplified by PCR using the corresponding primers listed in Supplementary Table S1, and then, respectively, inserted into vectors pBiFC-VN173 or pBiFC-VC155 (Addgene) for the expression of fusion proteins nsp-YFPN and nsp-YFPC. Human embryonic kidney (HEK) 293T cells were co-transfected in pairs with the constructs using calcium phosphate. Cells co-transfected with pBiFC-bjun-VN173 and pBiFC-bFosVC155 were used as positive control, and cells co-transfected with pBiFC-bjun-VN173 and pBiFC-bFos(deltaZIP)VC155 were set as negative control. The supernatant was replaced with fresh medium at 8 h post-transfection, and the fluorescence was examined 24 h post-transfection using a DM5000B microscope (Leica, Germany).

### Protein Expression in *Escherichia coli* and Purification

The expression and purification of nsp9-His (Liu et al., 2016), His-nsp7α and His-nsp7β (Chen et al., 2017) have been described previously. His-tagged tobacco etch virus protease (TEV-His) (Chen et al., 2017) was purified and used as a negative control. To express GST-nsp3 and GST-nsp5, the nsp genes without the transmembrane regions were amplified by over-lapping PCR and cloned into pGEX-2T vector. The exact expressed regions for each nsp are illustrated in **Table 1**. The GST-tagged proteins expressed in *Escherichia coli* BL21(DE3) strain were purified with GST-Bind Sepharose (CWBIO) in phosphate buffer (137 mM NaCl, 2.7 mM KCl, 10 mM Na_2_HPO_4_, and 2 mM KH_2_PO_4_ [pH 8.0]).

The nsp12 gene was amplified by PCR and inserted into vector pMAL-c4X (New England Biolabs). MBP-nsp12 was expressed in *E. coli* and purified using Amylose Resin (New England Biolabs) according to the manufacturer’s instruction.

To express Flag-tagged proteins, a pTriEx-flag vector was made by inserting annealed oligonucleotides Nco-flag (5′-CATGGATTACAAGGATGACGATGACAAGG-3′) and Bam-flag (5′-GATCCCTTGTCATCGTCATCCTTGTAATC-3′) into pTriEx1.1 (Novagene) between the *Nco*I and *Bam*HI sites. The nsp1α, nsp7α, nsp8 and nsp11 genes were amplified by PCR and cloned into pTriEx-flag for the expression of Flag-tagged nsps.

### Pull-Down Assay

His-tagged protein was mixed with GST/Flag tagged nsp. The mixture was incubated at 4°C for 2 h and mixed with PureProteome nickel magnetic beads (Millipore, United States). Proteins captured by the beads were eluted with elution buffer (50 mM sodium phosphate, 300 mM NaCl, and 300 mM imidazole [pH 8.0]), and then separated by SDS-PAGE and detected by Western blotting using tag antibodies (CWBIO). Similarly, MBP-nsp12 was mixed with GST/Flag/His tagged nsps, and the proteins co-purified with MBP-nsp12 using Amylose Resin were then separated by SDS-PAGE, stained with Coomassie brilliant blue or detected by Western blotting.

## Results

### Screening Nsp Interactions by Yeast Two-Hybrid

After the replicase polyprotein translation and processing in PRRSV infection, most of the non-structural proteins are believed to be involved in the RTC formation and participate in the virus replication and transcription processes. In order to investigate the associations between PRRSV nsps, we performed a yeast two-hybrid matrix screen with all the viral nsps except nsp6, which is only 16 amino acids in length. As both the AD- and BD- fused proteins need to enter the nucleus to activate the reporter expression, the putative transmembrane domains in nsp2, nsp3, and nsp5 were deleted to avoid detaining the transmembrane proteins with intracellular membranes (the peptides used in the study is listed in **Table 1**). The gene fragments of the tested nsps were cloned into both the pGBKT7 and pGADT7 vectors so that they could be tested as both prey and bait.

**FIGURE 1:**
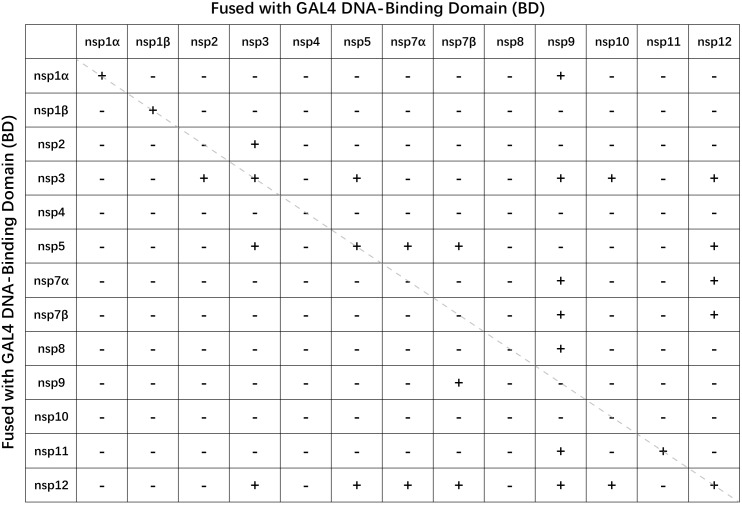
Analysis of PRRSV non-structural protein interactions by Y2H matrix screen. +, positive interaction; –, negative interaction.

The yeast two-hybrid screening resulted in 30 positive results out of the 169 tested protein combinations (**Figure 1** and Supplementary Figure S1), including previously reported interaction pairs of nsp1α–nsp1α (Sun et al., 2009), nsp1β–nsp1β (Xue et al., 2010), nsp2–nsp3 (Snijder et al., 2001), nsp7α–nsp9 (Chen et al., 2017), and nsp11–nsp11 (Shi et al., 2016). Among the positive interactions, 7 protein pairs (nsp2–nsp3, nsp3–nsp5, nsp3–nsp12, nsp5–nsp12, nsp7α–nsp12, nsp7β–nsp9, and nsp7β–nsp12) gave positive results no matter which one served as the bait or prey. Protein self-interactions were identified in 6 nsps (nsp1α, nsp1β, nsp3, nsp5, nsp11, and nsp12). Although only the interaction with nsp7β was detected for nsp9 as the prey, nsp9 fused with GAL4 BD domain was found to interact with 7 prey nsps (nsp1α, nsp3, nsp7α, nsp7β, nsp8, nsp11, and nsp12), supporting it as a prospective core component of the viral RTC. Nsp5 interacted with nsp7α and nsp7β only when it served as the prey. The interactions of nsp10 with nsp3 and nsp12 were positive when nsp10 acted as the bait. In Y2H, the bait is fused to the DNA binding domain and the prey is fused to the activation domain of the transcription factor. The different fusion partner and the relative position to the DNA strand may affect the folding and accessibility of the fusion proteins, which may result in the discrepancy in the interaction results for the nsps, respectively, acting as a bait or a prey. No positive interaction was identified for the main proteinase nsp4, whose associations with the replicase polypeptides might be very short-lived.

### BiFC Analysis of Nsp Interactions

**FIGURE 2:**
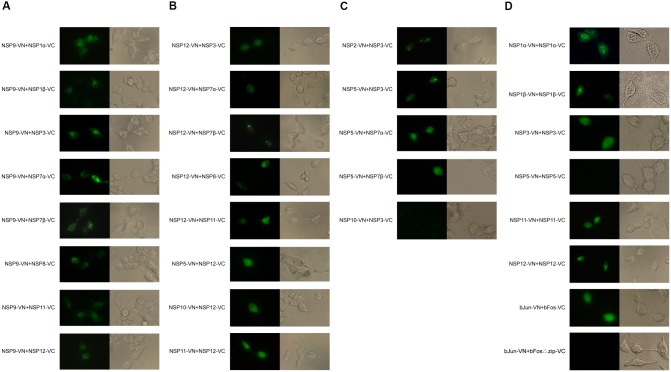
BiFC analysis of nsp interactions. Each nsp was fused to either the N- or C-terminal part of YFP. The fusion proteins were expressed in HEK 293T cells and YFP images in cells were visualized at 24 h post-transfection. **(A)** Interactions between nsp9 and other nsps. **(B)** Interactions between nsp12 and other nsps. **(C)** Interactions involving transmenbrane nsps. **(D)** Self-interactions and controls. Protein pair bJun-VN + bFos-VC was used as positive control, and bJun-VN + bFos(delta)zip-VC was negative control.

To eliminate potential false positive interactions between nsps identified by Y2H, the non-structural proteins were, respectively, fused to the N- or C-terminal part of enhanced yellow fluorescent protein (YFP), and protein-protein interactions were visualized under a fluorescence microscope. As shown in **Figure 2**, most of the combinations of nsps showed positive interactions in Y2H were confirmed in the BiFC assay, with the exception of nsp3–nsp10 (**Figure 2C**) and nsp5–nsp5 (**Figure 2D**) which did not yield observable fluorescence. Considering that the full-length transmembrane proteins were used in BiFC analysis but the transmembrane domains were deleted in Y2H assays, this result implied that the nsp5–nsp5 and nsp3–nsp10 interactions identified in Y2H using truncated nsp5 and nsp3 might be artifacts.

On the other hand, we observed three more interactions (nsp1β–nsp9, nsp8–nsp12, and nsp11–nsp12) in BiFC assays which were not detected by Y2H. BiFC enables direct visualization of protein interactions in living cells. It has been reported that it can detect weak interactions and the fluorophore reconstitution can occur at intermolecular distances over 7 nm (Hu et al., 2006; Kerppola, 2008). This may contribute to less steric hindrance and higher sensitivity in the detection of protein–protein interaction. In addition, Y2H takes place in the nucleus. For most PRRSV nsps which normally only localize in the cytoplasm, proteins detected interacting in the cytoplasm may be found to be non-interacting in the nucleus in Y2H.

Interacted nsp2–nsp3 and nsp3–nsp5 appeared as punctate foci in the perinuclear region of the cell, similar to the distribution of nsps observed in early infection (Li et al., 2012) and consistent with previous observations that expression of these transmembrane nsps could induce the formation of structures similar to the infection-induced DMVs (Snijder et al., 2001; van der Hoeven et al., 2016).

### Verification of Nsp Interactions by Pull-Down Assay

**FIGURE 3:**
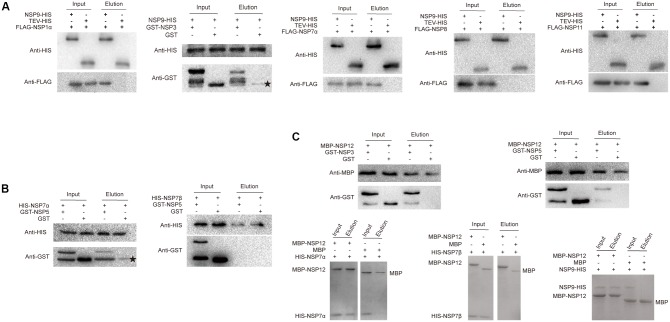
Pull-down analysis of nsp interactions. **(A)** Interaction of nsp9 with nsps using nsp9-His as bait. His-tagged tobacco etch virus protease (TEV-His) was used as a negative control bait in the assays. **(B)** Interaction of nsp5 with nsp7α/nsp7β. His-tagged nsp7α/nsp7β were used as bait. **(C)** Interaction of nsp12 with nsps using MBP-nsp12 as bait. In the protein pairs of nsp12–nsp7α, nsp12–nsp7β, and nsp12–nsp9, both the bait and prey were aboundantly purified so the protein bands separated by SDS-PAGE were directly visulized by Coomassie brilliant blue staining. The other nsp pairs in the input and output were detected by Western blot using tag antibodies. Asterisks indicate non-specific binding of GST.

By Y2H and BiFC, multiple interaction partners were detected for nsp9, nsp12 and transmembrane proteins nsp3 and nsp5, suggesting that these nsps might act as the core components in RTC formation. Pull-down assays were then performed to further validate these multiple interactions. Using nsp9-His as bait, the binding of nsp1α, nsp3, nsp7α and nsp11 to nsp9 were confirmed, but the nsp8–nsp9 interaction was not detected (**Figure 3A**). We observed that nsp8 was sensitive to degradation, therefore, the rapid degradation of the protein during the incubation of protein mixture could probably contribute to the failure in the detection of eluted nsp8 in pull-down assay.

To express the transmembrane proteins nsp3 and nsp5 in *E. coli*, the predicted hydrophobic transmembrane domains located in the middle of the polypeptides were deleted and the hydrophilic regions were linked by a GGGGS × 2 linker. The proteins were fused to both His-tag and GST-tag, however, the His-tagged proteins were predominantly expressed as insoluble inclusion bodies (data not shown) and only the GST-tagged nsp3 and nsp5 were moderately soluble and suit for pull-down assay. GST-nsp3 and GST-nsp5 were used as prey to detect their interactions with His-tagged nsp7α, nsp7β, nsp9, and MBP-tagged nsp12. Although a faint GST band as the control prey was sometimes observed to bind to the nickel magnetic beads (indicated by asterisks in **Figures 3A,B**) and cleaved GST was consistently detected in all the samples containing GST-nsp3/nsp5, the binding of GST-nsp3 to nsp9-his (**Figure 3A**) and GST-nsp5 to His-nsp7α (**Figure 3B**) was obvious. The interaction of GST-nsp5 to His-nsp7β was not detected (**Figure 3B**), probably due to the instability characteristics of nsp7β that was previously reported (Li et al., 2012) and also observed in our studies.

For pull-down analysis of the interaction of nsp12 with other nsps, MBP-tagged nsp12 was purified and used as bait. As shown in **Figure 3C**, the interactions of nsp12 with nsp3, nsp5, nsp7α, and nsp9 were clearly observed. Similar to the negative result for the interaction between GST-nsp5 and His-nsp7β, no His-nsp7β was detected binding to MBP-nsp12.

### PRRSV Nsp Interactome

**FIGURE 4:**
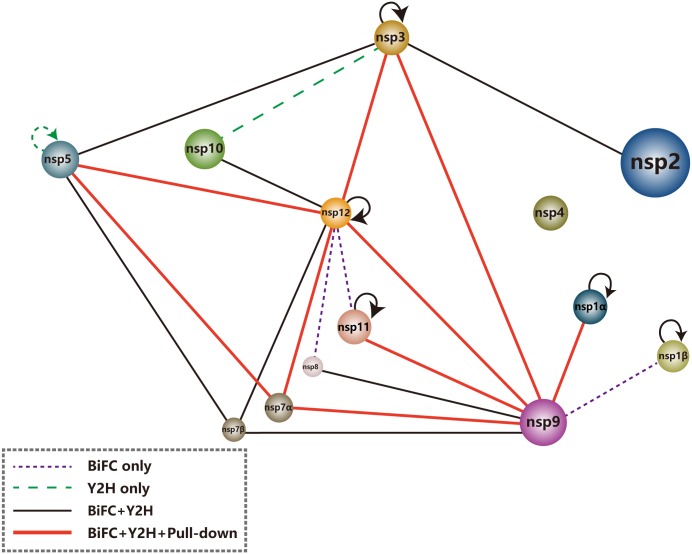
Schematic summary of nsp interactions identified by Y2H, BiFC, and pull-down assays.

Based on the Y2H, BiFC and pull-down results in this study, protein–protein interactions among PRRSV nsps are summarized in **Figure 4**. In this network, the viral RdRp (nsp9) was found to associate with multiple interaction partners including nsp1α, nsp1β, nsp3, nsp7α, nsp7β, nsp8, nsp11, and nsp12. Most of these interactions were screened by Y2H and verified both by BiFC and pull-down assays, except that the interaction with nsp1β was identified by BiFC and the associations with nsp7β and nsp8 were not detected by pull-down. Nsp7β and nsp8 were easily degradable and they were probably degraded during the *in vitro* assay. The multiple nsp associations of nsp9 validated the speculation of the RdRp as a core component of the viral RTC.

The second hub identified in the PRRSV nsp interactome was nsp12, whose interactions with nsp3, nsp5, nsp7α, and nsp9 were detected by all the three assays used in this study. The interactions of nsp12 with nsp7β, nsp10 and nsp12 itself were determined by both Y2H and BiFC. The nsp8–nsp12 and nsp11–nsp12 associations were only observed by BiFC.

Both transmembrane protein nsp2 and nsp5 interacted with transmembrane protein nsp3, and nsp3 could also bind to itself, suggesting that these three nsps might associate together to form a membrane-bound complex. Through the direct interactions of nsp3 with soluble proteins nsp9, nsp12 and maybe also nsp10, and the direct binding of nsp5 to nsp7α, nsp7β, and nsp12, the transmembrane proteins indirectly associated with most other nsps. As no interaction of nsp4 with other nsps was observed by Y2H, we did not further study it in BiFC and pull-down assays.

## Discussion

Like other positive-stranded RNA viruses, the replication and transcription of arteriviruses is compartmentalized by double-membrane vesicles (DMVs) (Pedersen et al., 1999; Snijder et al., 2001), where viral and some cellular proteins (van Hemert et al., 2008; Beura et al., 2011) form replication and transcription complexes (RTCs). The viral non-structural proteins are the major components of the RTCs and the transcription and replication of arteriviruses have been extensively studied, but the physical relationships among the viral proteins required for this process are mainly unknown. In this study, we investigated the network of interactions amongst the non-structural proteins that assemble into the PRRSV RTC.

**FIGURE 5:**
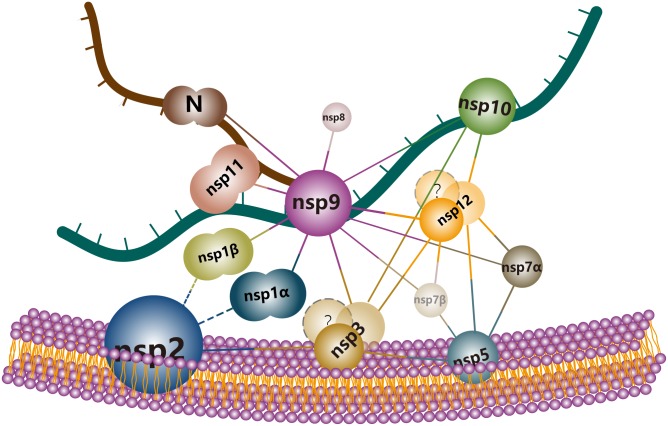
A model of PRRSV replication and transcription complex in DMVs. The three PRRSV transmembrane proteins (nsp2, nsp3, and nsp5) form a membrane-bound nsp2–nsp3(×n)-nsp5 scaffold for the support of other nsp components. Amongst the soluble nsps, nsp9 and nsp12 act as hubs in the nsp interaction network by interacting with multiple partners including both transmemebrane and soluble nsps. The interactions identified in our assays are linked with solid lines and the documented associations of nsp2 with nsp1α and nsp1β which were not detected in this study were indicated by dashed lines.

It has long been known that two transmembrane non-structural proteins (nsp2 and nsp3) of arterivirus equine arteritis virus (Fuchs et al., 1983) interact with each other and they are suffice to induce DMVs comparable to those formed during viral infection (Snijder et al., 2001). Recently, it was found that coexpression of nsp5 could result in the formation of more homogenous DMVs that were closer to what was observed upon EAV infection, suggesting that nsp5 played a regulatory role in modulating membrane curvature and DMV formation (van der Hoeven et al., 2016). Belonging to the same family Arterivirus, PRRSV has similar gene structure and closely related to EAV. It is conceivable that PRRSV transmembrane proteins nsp2, nsp3, and nsp5 may also recognize each other and act as the same roles in the DMV formation. By Y2H and BiFC, we found that both nsp2 and nsp5 interacted with nsp3, and nsp3 could also interact itself, indicating that the three PRRSV transmembrane proteins might form a membrane-bound nsp2-nsp3(×n)-nsp5 scaffold to support the formation of RTC for the viral replication and transcription (a model is illustrated in **Figure 5**). This model is consistent with previous finding that EAV nsp3 plays a key role in the remodeling of intracellular membranes (Posthuma et al., 2008). It is unclear if EAV nsp3 can also bind to itself, and further studies are needed to investigate whether PRRSV nsp3 is anchored on the intracellular membrane as a dimer or higher multimer.

As the viral RNA polymerase, arterivirus nsp9 has been thought as a core component in RTCs. Although previous studies have found that PRRSV nsp9 can interact with viral structural protein N (Liu et al., 2016), viral non-structural protein nsp7α (Chen et al., 2017), and some cellular proteins such as retinoblastoma protein (Dong et al., 2014) and DEAD-box RNA helicase 5 (Zhao et al., 2015), how nsp9 associates with other nsps is not understood. Here, we confirmed the nsp7α-nsp9 interaction and detected the direct binding of seven more nsps (nsp1α, nsp1β, nsp3, nsp7β, nsp8, nsp11, and nsp12) to nsp9. These data highlighted the central role of nsp9 within the RTC machinery.

During the infection of nidoviruses including arteriviruses and coronaviruses, the viruses produce the full-length genome using continuously synthesized minus-stranded RNA as template. They also generate a nested set of subgenomic mRNAs by a unique mechanism of discontinuous minus-strand RNA template synthesis (Sawicki and Sawicki, 1995). The relative abundance of the genomic and subgenomic RNAs is well controlled in the virus infection, and this is vital for efficient virus production (Nedialkova et al., 2010). It is unclear how the RTC fine-tunes these various RNA synthesis processes. The association of specific replicase subunits with the RNA polymerase may regulate the balance between replication and transcription. EAV nsp1 has been identified as a “transcription factor” for the RdRp switch from continuous into discontinuous mode (Nedialkova et al., 2010). Our interactome analyses detected the direct binding of nsp1α to nsp9 by all the three assays, consisting with the previous observation that the N-terminal zinc finger domain of nsp1 was important for this function (Tijms et al., 2001; Nedialkova et al., 2010). Mutations in other nsp1 domains were also found to strongly influence transcription (Tijms et al., 2001; Nedialkova et al., 2010). We did not detect the interaction of nsp1β to any nsps other than itself in Y2H screening, but nsp1β–nsp9 interaction was clearly observed by BiFC. It has been observed that nsp1α and nsp1β also directly interact with nsp2 by immunoprecipitation (Song et al., 2016). Our Y2H assay showed negative results for these two pairs, which might be false negative results due to the relatively large sizes of the protein pairs, and further studies are needed to explore this.

An interactome study of SARS-coronavirus nsps demonstrated that viral key enzymes encoded by ORF1b, including the RNA helicase (nsp13), 3′-5′exonuclease (nsp14), endonuclease (nsp15) and putative methyltransferase (nsp16), all bound to the RdRp (nsp12), leading to the hypothesis of the existence of a proofreading mechanism for the faithful replication and transcription of the viral long RNA genome (Imbert et al., 2008). For PRRSV, we observed the interaction of the endonuclease (nsp11) to the RdRp (nsp9), but did not detect the direct association of the RNA helicase (nsp10) with nsp9. However, our data revealed an indirect interaction of PRRSV nsp10 with nsp9 via nsp12 (**Figure 4**).

Besides nsp9, the protein–protein interaction analyses also identified multiple interaction partners (nsp3, nsp5, nsp7α, nsp7β, nsp8, nsp9, nsp10, nsp11, and nsp12 itself) for nsp12, the structure and function of which is still unknown. Using high throughput proteomics, we have found that PRRSV nsp12 interacts with many cellular proteins with high probability (Dong et al., 2016). Among the cellular proteins, HSP70 is recruited by nsp12 to maintain the protein’s stability and benefit the viral replication. Another study have shown that nsp12 induces the phosphorylation of STAT1 at serine 727 and the expression of proinflammatory cytokines IL-1β and IL-8 (Yu et al., 2013). In a most recent report, nsp12 is documented to be able to induce the stabilization of karyopherin alpha6 which is required for the nuclear translocation of nsp1β and the PRRSV replication (Yang et al., 2018). These previous data have demonstrated the involvement of PRRSV nsp12 in the virus infection by modulating cellular factors. The identification of multiple interactions of nsp12 with viral nsps in this study suggests that nsp12, which probably exists as a homologous dimer or multimer by self-interaction, should also play an important role in the viral RNA synthesis process as a crucial component of the RTC.

## Conclusion

We investigated the potential interactions between PRRSV nsps, which may be involved in the formation of viral replication and transcription complex. Our data confirmed most of the documented interactions among PRRSV nsps, and identified a number of novel associations, which may provide valuable clues for future researches such as the viral transcription and replication mechanism and antiviral drug design.

## Author Contributions

HC and XX conceived and designed the research. HN, JL, MT, SD, JT, and LL performed the Y2H screening. HN, JL, and JT conducted the BiFC assays. HN and JL carried out pull-down assays. HC, XX, HN, and JL analyzed the data. HC wrote the manuscript.

## Conflict of Interest Statement

The authors declare that the research was conducted in the absence of any commercial or financial relationships that could be construed as a potential conflict of interest.
