# Supplementary material for: The Network of Interactions Among Porcine Reproductive and Respiratory Syndrome Virus Non-structural Proteins
Source: Front Microbiol. 2018 May 14;9:970. doi: 10.3389/fmicb.2018.00970 (PMC5960727; doi:10.3389/fmicb.2018.00970)
Supplement: Supplementary file 1 [file Data_Sheet_1.docx]

***Supplementary Material***

**The Network of Interactions among Porcine Reproductive and Respiratory Syndrome Virus Non-structural Proteins**

**Hao Nan^1^, Jixun Lan^1^, Mengmeng Tian^1^, Shan Dong^1^, Jiao Tian^1^, Long Liu^1,2^, Xiaodong Xu^1^, Hongying Chen^1, 3*^**

*** Correspondence:** Corresponding Author: [chenhy@nwafu.edu.cn](mailto:chenhy@nwafu.edu.cn)

**Supplementary Tables**

Supplementary Table 1**.** PCR primers used in this study for Y2H, BiFC and pull-down assays.

| **Primer** | **Sequence (5′-3′)** |
| --- | --- |
| **Y2H** |  |
| NdeINSP1α-F | GGAATTCCATATGATGTCTGGGATACTTGATCGG |
| EcoRINSP1α-R | TCGTGAATTCCATAGCACACTCAAAAGGGC |
| NdeINSP1β-F | GGAATTCCATATGGCTGACGTCTATGATATTGG |
| EcoRINSP1β-R | TAATGAATTCACCGTACCATTTGTGACTG |
| NdeINSP2-F | GGAATTCCATATGGCTGGAAAGAGAGCAAGG |
| BamHINSP2-R | CTAAGGATCCGGGTAAGTCAGCACCACC |
| NdeINSP3OD1-F | GGAATTCCATATGTATGTAACTGCAGTGGGGTC |
| BamHINSP3OD1-R | CATAGGATCCCACAAGTGCTGTCAAGGGC |
| NdeINSP3OD2-F | GGAATTCCATATGCGTTATACTAATGTTGTTGGTC |
| BamHINSP3OD2-R | GAATGGATCCCTCAAGAAGGGACCCAAG |
| EcoRINSP4-F | TAATGAATTCGGCGCTTTCAGAACTCGA |
| BamHINSP4-R | TAATGGATCCTTCCAGTTCGGGTTTGGC |
| NdeINSP5OD1-F | GGAATTCCATATGGGACATGCCTGGACG |
| BamHINSP5OD1-R | CACGGGATCCTCTGTTCCTGTTAAGAG |
| NdeINSP5OD2-F | GGAATTCCATATGGCAACTCAAGGGCACCCG |
| BamHINSP5OD2-R | ATTAGGATCCCCGAGGCAGGAAGGCATAG |
| EcoRINSP7α-F | TAATGAATTCTCGCTGACTGGTGCCCTC |
| BamHINSP7α-R | TAATGGATCCCTCCAGAACTTTCGGTGG |
| EcoRIHINSP7β-F | TGAAGAATTCAATGGTCCCAACGCCTGG |
| BamHINSP7β-R | TAATGGATCCTTATTGGTTTTTTTTGCTC |
| EcoRINSP8-F | TAATGAATTCGCTGCGAAGCTTTCCGTG |
| BamHINSP8-R | TAATGGATCCGCAGTTTAAACACTGCTC |
| BamHINSP9-F | TAATGGATCCAAGGAGCAGTGTTTAAACTG |
| PstINSP9-R | TAATCTGCAGCTCATGATTGGATCTGAGT |
| BamNSP9-F | ACGCGGATCCCAggagcagtgtttaaactgct |
| XhoNSP9-R | ACCGCTCGAGctcatgattggatctgagtt |
| NdeINSP10-F | GGAATTCCATATGGGGAAGAAGTCC |
| BamHINSP10-R | TAATGGATCCTTCCAGATCTGC |
| EcoRINSP11-F | TAATGAATTCGGGTCGAGCTCCCCGCTC |
| BamHINSP11-R | TAATGGATCCTTCAAGTTGAAAATAGGC |
| EcoRINSP12-F | TAATGAATTCGGCCGCCATTTCACCTGGT |
| BamHINSP12-R | TAATGGATCCTCAATTCAGGCCTAAAGTTG |
| **BiFC** |  |
| N-HindNSP1α-F | GCCGAAGCTTatgtctgggatacttgatcg |
| N-XbaNSP1α-R | CGTATCTAGAcatagcacactcaaaagggc |
| HindNSP1β-F | GCCGAAGCTTgctgacgtctatgatattgg |
| XbaNSP1β-R | CGTATCTAGAaccgtaccatttgtgactgc |
| N-NotINSP2-F | TAATGCGGCCGCTGCTGGAAAGAGAGCAAGG |
| N-EcoRVNSP2-R | TAGAGATATCACTCCTCCCGAGGGCTTGG |
| N-HindIIINSP3-F | GCCGAAGCTTTATGTAACTGCAGTGGGGTC |
| N-XbaINSP3-R | CGTATCTAGACTCAAGAAGGGACCCAAG |
| N-HindNSP5-F | TTGTAAGCTTGGAGGCCTCTCCACCGTC |
| N-SalINSP5-R | TTGTGTCGACCTCGGCAAAGTATCGCAAG |
| N-SalINSP9-F | TTGTGTCGACGGAGCAGTGTTTAAACTG |
| N-XhoINSP9-R | TTGTCTCGAGCTCATGTTAGGATCTGAG |
| N-HindIIINSP10-F | TTGTAAGCTTGGGAAGAAGTCCAGAATG |
| N-SalINSP10-R | TTGTGTCGACTTCCAGATCTGCACAAATG |
| N-EcoRINSP11-F | TATAGAATTCAGGGTCGAGCTCCCCGCTC |
| N-SalINSP11-R | GCCGGTCGACTTCAAGTTGAAAATAGGC |
| N-EcoRINSP12-F | TAATGAATTCGGCCGCCATTTCACCTGG |
| N-SalINSP12-R | GCCGGTCGACTCAATTCAGGCCTAAAGTTG |
| C-EcoRINSP1α-F | GCCGGAATTCACATGTCTGGGATACTTG |
| C-XhoINSP1α-R | TATACTCGAGACATAGCACACTCAAAAGG |
| C-EcoRINSP1β-F | GCCGGAATTCACGCTGACGTCTATGATATTG |
| C-XhoINSP1β-R | TATACTCGAGAACCGTACCATTTGTGAC |
| C-EcoRINSP3-F | TATAGAATTCACGGCCCGCACCTCATTGC |
| C-XhoINSP3-R | TATACTCGAGACTCAAGAAGGGACCCAAG |
| C-EcoRINSP5-F | TAACGAATTCAGGGACATGCCTGGACG |
| C-XhoINSP5-R | TACACTCGAGACCGAGGCAGGAAGGCATAG |
| C-EcoRINSP7α-F | TAATGAATTCTCTCGCTGACTGGTGCCCTC |
| C-XhoINSP7α-R | GCCGCTCGAGACTCCAGAACTTTCGGTGG |
| C-EcoRINSP7β-F | TAATGAATTCTCAATGGTCCCAACGCCTGG |
| C-XhoINSP7β-R | GCCGCTCGAGCTTCCCATTGGTTTTTTTTGC |
| C-EcoRINSP8-F | TATAGAATTCACGCTGCGAAGCTTTCCGTG |
| C-XhoINSP8-R | TATACTCGAGAGCAGTTTAAACACTGCTC |
| C-EcoRINSP11-F | GCCGGAATTCACGGGTCGAGCTCCCCGCTC |
| C-XhoINSP11-R | GCTGCTCGAGATTCAAGTTGAAAATAGGC |
| C-EcoRINSP12-F | TAATGAATTCGGCCGCCATTTCACCTGG |
| C-XhoINSP12-R | GCCGCTCGAGTCAATTCAGGCCTAAAGTTG |
| **Pull-down** |  |
| NcoINSP1α-F | TTGTCCATGGCCTCTGGGATACTTGATCGGTG |
| XhoINSP1α-R | TTGTCTCGAGCATAGCACACTCAAAAGG |
| BamHINSP3OD1-F | ACGCGGATCCTATGTAACTGCAGTGGGGTC |
| G4SNSP3OD1-R | GCTCCCGCCACCTCCAGAGCCTCCGCCACCCACAAGTGCTGTCAAGGGC |
| G4SNSP3OD2-F | GGAGGTGGCGGGAGCGGCGGTGGAGGGTCTCGTTATACTAATGTTGTTGGTC |
| EcoRINSP3OD2-R | ACCGGAATTCCTCAAGAAGGGACCCAAG |
| BamHINSP5OD1-F | ACGCGGATCCGGACATGCCTGGACG |
| G4SNSP5OD1-R | GCTCCCGCCACCTCCAGAGCCTCCGCCACCTCTGTTCCTGTTAAGAG |
| G4SNSP5OD2-F | GGAGGTGGCGGGAGCGGCGGTGGAGGGTCTGCAACTCAAGGGCACCCG |
| EcoRINSP5OD2-R | ACCGGAATTCCCGAGGCAGGAAGGCATAG |
| NcoINSP7α-F | TTGTCCATGGTCGCTGACTGGTGCCCTC |
| XhoINSP7α-R | TAATCTCGAGTCACTCCAGAACTTTCGGT |
| NcoINSP8-F | TTGTCCATGGCTGCGAAGCTTTCCGTG |
| XhoINSP8-R | TTGTCTCGAGGCAGTTTAAACACTGCTCC |
| NcoINSP11-F | TTGTCCATGGGGTCGAGCTCCCCGCTCC |
| XhoINSP11-R | TTGTCTCGAGTTCAAGTTGAAAATAGGC |
| BamHINSP12-F | TCAGGGATCCGGCCGCCATTTCACCTG |
| HindIIINSP12-R | TGCCAAGCTTTCAATTCAGGCCTAAAGTTG |

**Supplementary Figures**

**
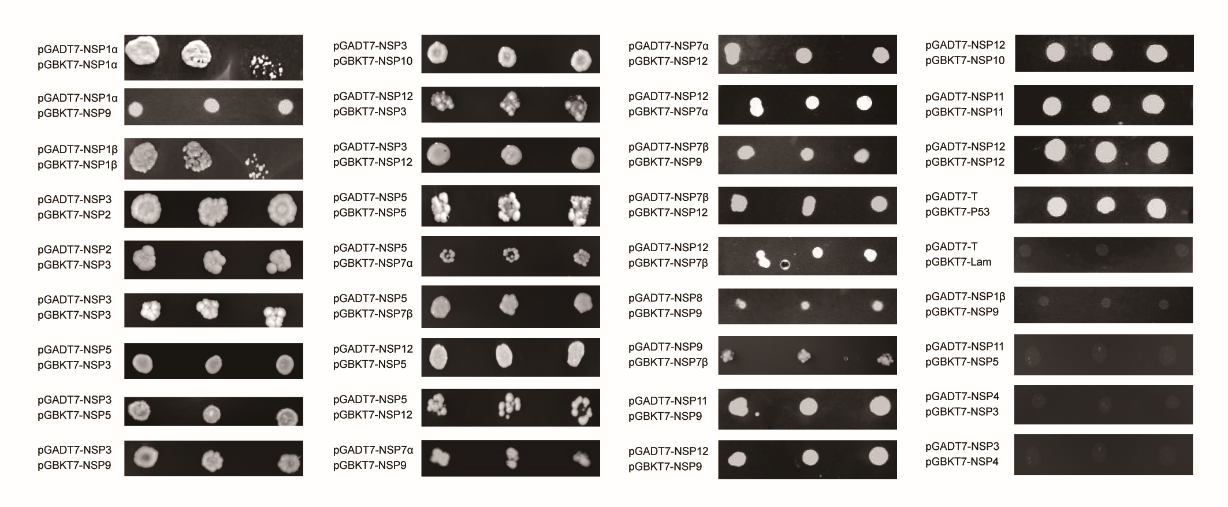
**

Supplementary Figure 1. Analysis of PRRSV nonstructural protein interactions by yeast two-hybrid. The Y2H screening resulted in 30 positive results as shown. The yeast cells co-transformed with pGBK7-P53 and pGADT7-T were used as the positive controls, and the yeast cells harboring pGBKT7-Lam and pGADT7-T were served as the negative control.
